# Comparative Analysis of Selective Bacterial Colonization by Polyethylene and Polyethylene Terephthalate Microplastics

**DOI:** 10.3389/fmicb.2022.836052

**Published:** 2022-02-02

**Authors:** Yuhao Song, Baoxin Zhang, Lianwei Zou, Feng Xu, Yaqi Wang, Shaoqi Xin, Yang Wang, Hongyuan Zhang, Ning Ding, Renjun Wang

**Affiliations:** College of Life Sciences, Qufu Normal University, Qufu, China

**Keywords:** polyethylene, polyethylene terephthalate, microplastic-attached biofilm, freshwater, biodiversity

## Abstract

In this study, we report the biodiversity and functional characteristics of microplastic-attached biofilms originating from two freshwater bacterial communities. Even though the microplastic-biofilm (MPB) diversities are mostly determined by original bacteria instead of microplastic types, the results from 16S rRNA amplicon sequencing still showed that the dynamic biofilm successions on different microplastics were highly dissimilar. Furthermore, the analysis of biomarkers indicated distinct bacterial species with significant dissimilarities between different MPBs, which further determined the associated functions. The co-occurrence networks showed distinct interconnective characteristics in different MPBs: The structure of MPB incubated in the lake water sample was more robust under environmental stresses, and bacteria in the tap water MPB interacted more cooperatively. Regarding this cooperative interaction, the analysis of functional prediction, in this study, also showed that more symbionts and parasites colonized on microplastics in the tap water than in the lake water. Moreover, it was suggested that MPBs were more easily formed in the tap water sample. The overall results revealed significant dissimilarities in bacterial diversity, succession, and associated functions between MPBs, in which bacterial species with specific functions should be taken seriously.

## Introduction

Plastic is a common polymeric material worldwide owing to its excellent performance (e.g., durability, anticorrosion, and plasticity) and relatively low cost, and it has already played indispensable roles in all walks of life. With an increasing demand for diverse types of plastic, its global production increased continuously from 1.7 to almost 360 million tons during the last 70 years (PlasticsEurope, 2019), consequently leading to a large amount of plastic waste that has been disposed into the environment. Among these disposed plastics, plastic components of polyethylene (PE), polyethylene terephthalate (PET), and polypropylene (PP) are commonly produced and used, and they account for 90% of plastic production, along with polystyrene (PS) and polyvinyl chloride (PVC; Andrady and Neal, 2009; Alimi et al., 2018). Generally, these disposed plastics exhibit poor degradability in natural environments, primarily resulting in the formation of microplastics of different sizes (<5 mm) and shapes (e.g., fibers and fragments) by physical (e.g., mechanical wear), chemical (e.g., photooxidation), and biological processes (Hidalgo-Ruz et al., 2012; De Sa et al., 2018; Rachman, 2018; Shabbir et al., 2020).

Regarding aquatic ecosystems, microplastics are extensively distributed in most types of marine and freshwater environments (Klein et al., 2015; Ivleva et al., 2017; Di and Wang, 2018), and they have even been detected in preserved areas and polar regions in recent years (Claessens et al., 2013; De Sa et al., 2018). A number of studies have indicated that these microplastics exhibit toxicological effects on various aquatic organisms through uptake and accumulation; further, they pose a potential threat to human health by transmission along the food web (Cole et al., 2015; Sussarellu et al., 2016; Connors et al., 2017; Chapron et al., 2018; Ogonowski et al., 2018). In addition, microplastics have been revealed to be indirect harmful factors, which may serve as vectors that selectively enrich aquatic pathogens to new environments compared with natural substrates (e.g., rock and leaf) and could provide footholds for pathogens to invade aquatic organisms (e.g., coral; Lamb et al., 2018; Wu et al., 2019). Through long-term exposure and migration by water flow in aquatic environments, microplastics can be potential carbon sources that gradually release organic compounds into the water phase, thus contributing to the prolonged survival and growth of surrounding bacterial communities and the selective colonization of microplastic-attached biofilms that are distinct from other natural substrates (Tomboulian et al., 2004; Stern and Lagos, 2008; De Tender et al., 2015; Rummel et al., 2017).

When microplastics were disposed into the aquatic environments, the microplastic-attached biofilms were rapidly assembled and could be observed within 2 weeks, and their community structures are generally much different from that of the planktonic bacterial assemblages (McCormick et al., 2014, 2016; De Tender et al., 2017). Therefore, it has been suggested that microplastics are unique niches that colonize bacteria containing specific structural and functional patterns (Zettler et al., 2013; Miao et al., 2019). For example, recent studies have identified polymeric materials as potential vectors that carry aquatic (opportunistic) pathogens and harmful algae to remote environments (Viršek et al., 2017; Arias-Andres et al., 2018). More importantly, horizontal gene transfer may occur between biofilm and planktonic bacteria that mediates the transformation of several important genes such as antibiotic resistant genes (Bengtsson-Palme and Larsson, 2015; Li et al., 2015; Martinez et al., 2015; Arias-Andres et al., 2018). Until now, most studies have focused on two perspectives in terms of the comparison of microplastic-attached vs. planktonic bacteria and bacteria on natural vs. microplastic substrates (De Tender et al., 2015, 2017; Chae and An, 2017; Jiang et al., 2018; Miao et al., 2019; Wu et al., 2019), but the knowledge of biodiversity and functional characteristics of microplastic-attached biofilms exposed to different freshwater sources is still insufficient.

Thus, in this study, two commonly used plastic components, PE and PET materials were chosen as the microplastics tested for bacterial colonization, and two freshwater bacterial communities, one from lake water and one from tap water, were selected as the bacterial source of biofilms. 16S rRNA gene amplicon sequencing was performed to determine the bacterial diversities of biofilms, and bioinformatic tools were adopted to investigate the potential functions of each biofilm on microplastics. This study aims to decipher microplastic-attached biofilms with respect to (i) bacterial diversities of microplastic-biofilms (MPBs) in freshwater habitats and (ii) the associated functional patterns.

## Materials and Methods

### Sample Collection and Preparation of Microplastics

In order to culture the MPBs in this study, two freshwater bacterial communities, one originating from tap water and the other from lake water, were, respectively, collected from the in-house plumbing system (keep the water flowing for 5 min before sampling) of the drinking water distribution network at Qufu Normal University and Nansi Lake, Shandong Province, East China. Nansi Lake is the largest freshwater lake in northern China and its branch rivers flow through many cities as crucial drinking water sources. Here, each sample in triplicate was placed in pretreated carbon-free glass bottles (the method of carbon-free glassware preparation is described in the Supplementary Material) and then stored at 4°C during transportation and processed within 24 h. The main water quality parameters of two freshwater samples were determined and are listed in Supplementary Table 1. Prior to the related experiments, samples were prefiltered through 10-μm membrane filters to remove large organisms and particles.

Polyethylene and PET were chosen as the microplastics tested in this study. The PET material was purchased from Sigma-Aldrich Co., Ltd. (Shanghai, China) with a columned form (diameter: ~3.5 mm, length: ~4.5 mm), and the PE material was processed from a drinking water pipe (poly-ethylene silane cross-linked, PE-Xb) with a lamellar form (length: ~4.0 mm, width: ~3.5 mm, and thickness: ~1.5 mm). The selection of millimeter-sized PE and PET materials aims to provide sufficient surface area for the bacterial colonization that could reflect the biodiversity and interconnections of MPBs more comprehensively. Prior to the test, the microplastic particles used in the subsequent experiment were washed three times with ultrapure water and then autoclaved (120°C for 30 min) to avoid any contamination.

### Experiment of Biofilm Incubation on Microplastics

To investigate the bacterial growth and diversity of biofilms originating from two freshwater types on microplastics, 30 particles of each microplastic (~750 mg) in triplicate were, respectively, added into 1-L carbon-free glass bottles (Schott Duran, Germany) containing 500 ml freshwater samples. Subsequently, the biofilms on microplastic particles were incubated at 25°C for 30 days. Meanwhile, the original lake and tap water samples without the addition of microplastics (i.e., the planktonic bacteria in the freshwater samples) in triplicate were set, respectively, as the experimental controls. During this experiment, all samples for the biofilm incubation were supplemented with equivalent volume of their sterile sampling water regularly to maintain stable abiotic conditions. Three particles of PE and PET microplastics were taken at regular intervals (every 10 days) for further DNA extraction.

### DNA Extraction, 16S rRNA Amplicon Sequencing, and Sequence Processing

The PE and PET microplastics for biofilm-DNA extraction were firstly rinsed three times with sterilized phosphate buffer solution to remove the free-living bacteria. The DNA extraction of biofilms on microplastics was then processed using the PowerBiofilm® DNA isolation Kit (MoBio Laboratories, Carlsbad, CA, United States) in accordance with the manufacturer’s protocol. The biofilm-DNA obtained was inspected through 2.0% agarose gel electrophoresis and quantified with a Qubit® 2.0 Fluorometer (Invitrogen, Life technologies, Thermo Fisher Scientific Inc., United States) using a Qubit® dsDNA BR Assay kit (Invitrogen, Life technologies, Thermo Fisher Scientific Inc., United States). Prior to 16S rRNA amplicon sequencing, biofilm samples and biofilm sample groups were classified and named according to the freshwater source, types of microplastic, and incubation time (e.g., “L-PE-10” represents a biofilm sample originating from lake water on PE particles after incubation for 10 days; “L-PE” represents the biofilm sample group that includes samples “L-PE-10,” “L-PE-20,” and “L-PE-30”; and “L-PE/PET-0” and “T-PE/PET-0” represent the planktonic lake and tap water bacteria).

Gene amplification and sequencing were conducted using an Illumina platform with HiSeq2500 paired-end 250 bp mode as described previously (Liu et al., 2014). Primer pairs of 341 F (5′-CCTACGGGNGGCWGCAG-3′) and 806 R (5′-GGACTACHVGGGTATCTAAT-3′) were chosen to amplify the V3–V4 hypervariable region of bacterial and archaeal 16S rRNA genes. The raw sequence data were carried out using the QIIME pipeline (version 1.9.0; Caporaso et al., 2010). The quality of raw reads was controlled by excluding high-nitrogen (>20% of total reads) and low-quality (quality score ≥ 20) reads. Then, the two pair-end sequencing data were merged *via* FLASH v1.2.11 and quality filtered (Bokulich et al., 2013). The ultimate sequences were obtained by removing chimeras through comparison with the Gold database r20110519 using the UCHIME algorithm (Edgar et al., 2011). For clustering analysis, these sequences were clustered into operational taxonomic units (OTUs) by 97% similarity in USEARCH v7.0.1090 (Edgar, 2013), and taxonomy assignments were conducted using the SILVA database as the reference (Quast et al., 2013). OTUs with no annotation or archaea-related annotation were excluded. The Ribosomal Database Program classifier was used for species annotation to all OTUs at a confidence limit of 0.8–1.

### Statistical Analysis and Functional Gene Prediction

Non-metric multidimensional scaling (NMDS) was performed for visualization of biofilm bacterial community similarities based on the Bray–Curtis dissimilarity using the Vegan package in R (Philip, 2003).

The linear discriminant analysis (LDA) effect size (LEfSe) analysis was performed for visualization of specifically major biofilm bacterial clusters between different sample groups using LEfSe v1.0 software (Nicola et al., 2011). Kruskal–Wallis and Wilcoxon sum tests between sample groups were used for the selection of specifically major biofilm bacterial clusters in each sample group, and the relative importance of each bacterial cluster was then evaluated through LDA. In this study, bacterial clusters with an LDA score ≥ 4 were selected, because they have strong relative importance in each biofilm sample group.

Two aspects of the potential functions of each biofilm sample group were predicted using corresponding bioinformatic tools. The metabolic functions based on the Kyoto Encyclopedia of Genes and Genomes (KEGG) pathway were analyzed using Tax4Fun (Aßhauer et al., 2015), and ecological functions were assessed using the functional annotation of prokaryotic taxa (FAPROTAX; Louca et al., 2016).

Correlation analysis between variables was performed through linear pairwise Pearson correlation in the SPSS software, and the dissimilarities of alpha diversity indexes and relative abundance of functional genes between sample groups were evaluated using a paired-sample *t*-test in the SPSS software.

## Results and Discussion

### Phylogenetic Diversity of Bacterial Communities in MPBs

16S rRNA amplicon sequencing was performed for the comparative analysis of MPB diversities originating from lake and tap water samples. All of the MPB DNA samples reached sufficient sequencing depths (Good’s coverage values > 0.99) to ensure the accurate results (Supplementary Table 2).

**Figure 1:**
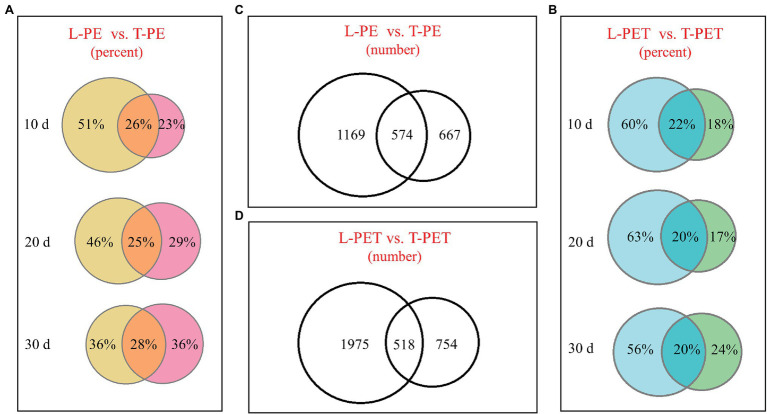
Venn diagrams showing the operational taxonomic units (OTUs) number or percentage of microplastic-biofilms (MPBs). **(A,B)** The OTUs percentage of polyethylene (PE) and polyethylene terephthalate (PET) biofilms incubated with lake and tap water at *t* = 10, 20, and 30 days; **(C,D)** the OTU number of MPB sample groups.

Biofilm samples at *t* = 10, 20, and 30 days were drawn for the evaluation of changes in bacterial community during incubation. Along with increasing incubation time, the overlapped bacterial OTUs between lake and tap water MPBs were relatively constant (no more than 30%), whereas the intrinsic bacterial OTUs showed distinct patterns (Figures 1A,B; Supplementary Table 3). Regarding the PE material, the intrinsic OTU number of lake water PE-biofilms gradually decreased, which was the converse of that of tap water PE-biofilms. However, the changes in intrinsic OTUs of L-PET and T-PET were irregular (Figures 1A,B; Supplementary Table 3). Moreover, the overall OTU number of lake water MPBs was much higher than that of tap water MPBs, regardless of PE and PET materials (Figures 1C,D). These results suggested that different microplastics serve as new vectors for different species sorting during biofilm growth and succession, thus resulting in diverse bacterial survival strategies of MPBs and varying degrees of reduced ability to withstand external stresses and maintain their fundamental activities compared to the original bacterial communities (Girvan et al., 2005; Philippot et al., 2013; Miao et al., 2019).

**Figure 2:**
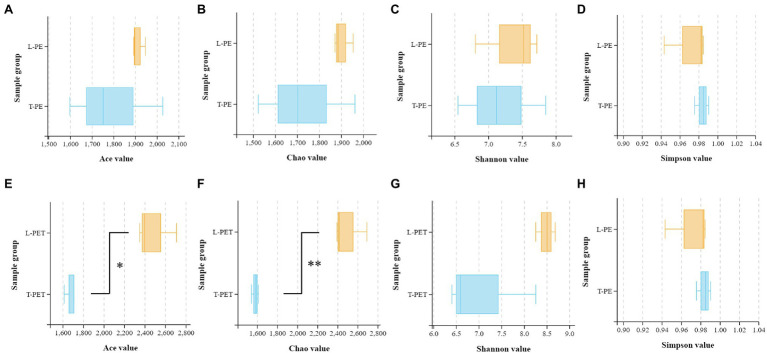
Box plots showing the Ace **(A,E)**, Chao **(B,F)**, Shannon **(C,G)**, and Simpson **(D,H)** indexes related to alpha diversity of different biofilm sample groups (**A-D**: L-PE vs. T-PE; E-H: L-PET vs. T-PET). The asterisks (* and **) represent significant (*p* < 0.05 and *p* < 0.01) dissimilarities between two MPB sample groups.

In this study, the alpha diversity indices (Chao, Ace, Shannon, and Simpson) were applied for the evaluation of biofilm bacterial richness and evenness on PE and PET materials (Figures 2A-H; Supplementary Table 4). As mentioned above, it can also be demonstrated that there were fewer diverse bacterial species of MPBs from tap water than lake water through the comparison of Chao and Ace indices, in which the values of T-PE and T-PET were lower than those of L-PE and L-PET (Figures 2A,B,E; Supplementary Table 4), suggesting that MPBs have stronger resistance and resilience to perturbation in lake water than in tap water (Girvan et al., 2005; Philippot et al., 2013). More importantly, the Chao and Ace indices exhibited significant dissimilarities (*p* < 0.01 and *p* < 0.05) between L-PET and T-PET (Figures 2E,F), indicating that the changes in bacterial richness for lake and tap water biofilms were significantly different on PET material over the course of 30 days. Considering the Shannon and Simpson indices, the bacterial evenness of lake water MPBs was higher than that of tap water MPBs, and yet there were no statistical dissimilarities.

**Figure 3:**
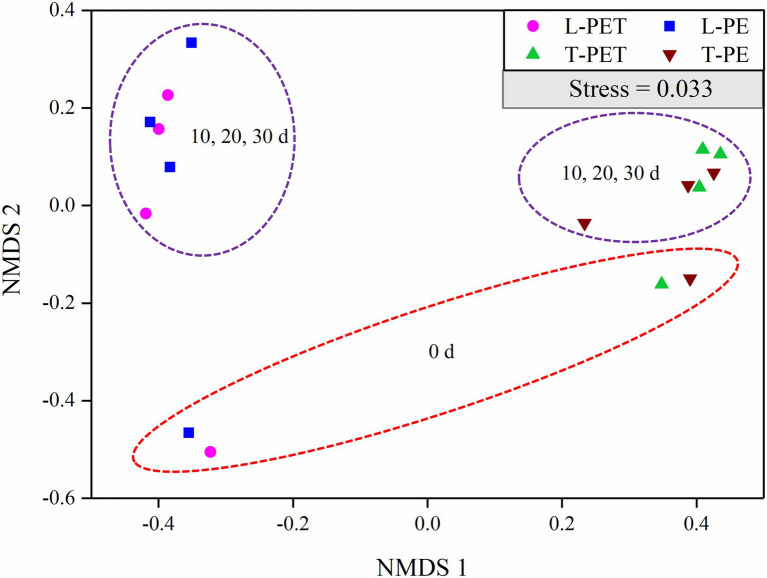
Non-metric multidimensional scaling (NMDS) of biofilm bacterial communities originating from lake and tap water on microplastics (represented by OTUs) calculated with Bray–Curtis dissimilarity between samples. In this plot, points that are closer together represent more similar bacterial communities to each other than those further away. A low stress value indicates a robust diagram.

In addition, the NMDS analysis was performed to compare the bacterial community structure in MPBs. Firstly, as shown in Figure 3, the bacterial community structure of MPBs was much different from that of the planktonic bacterial communities (i.e., the experimental controls; Supplementary Figure 1), especially for the lake water samples. This suggested that more bacterial species in tap water were selectively attached to microplastics, and consequently the bacterial community structures of MPBs were more closely related to the original bacterial communities than lake water. Secondly, there were no dramatic differences in biofilm bacterial community structure between PE and PET materials in the same water sample (Figure 3), indicating that in this study, the original bacterial communities in freshwater habitats, instead of microplastic types, undoubtedly determined the associated MPBs; this result is consistent with those of previous studies (Miao et al., 2019; Wu et al., 2019; Wang et al., 2020). However, the alpha diversity indices, including Ace, Chao, and Shannon values, were significantly dissimilar between L-PE and L-PET, which suggested distinct bacterial successions of MPBs in PE and PET materials in lake water during the 30 days of incubation (Supplementary Figure 2).

Interestingly, the phylum-level analysis of MPBs revealed that the relative abundance of predominant biofilm bacteria was dependent on microplastic types, of which the phyla Proteobacteria and Planctomycetes were the most abundant bacteria on PE and PET materials, respectively (Supplementary Figure 3; Supplementary Table 5). These two phyla of biofilm bacteria commonly attach to diverse types of polymeric materials (Miao et al., 2019; Wu et al., 2019; Li et al., 2020; Wang et al., 2020). Proteobacteria are critical for the community stability of MPBs and gene transfer among the biofilm bacterial species (Wu et al., 2019; Wang et al., 2020), and Planctomycetes was reported to be closely related to ammonium oxidation (Mohamed et al., 2010). However, this “microplastic-dependent” pattern was not applicable to the predominant biofilm bacteria at the class and genus levels. Of the subclasses of Proteobacteria, *Gammaproteobacteria*, which are repeatedly reported to be the predominant bacterial class in MPBs in both freshwater and seawater environments (Wu et al., 2019; Wang et al., 2020), accounted for the highest proportion of MPB bacteria in this study, followed by *Alphaproteobacteria* (except for L-PE; Supplementary Figure 3; Supplementary Table 5). In addition, *Aquabacterium* (Proteobacteria), *Fimbriiglobus* (Planctomycetes), and *Gemmata* (Planctomycetes) were major bacterial genera of tap water MPBs, whereas *Pirellula* (Planctomycetes) and *Chthoniobacter* (Verrucomicrobia) were generally predominant bacterial general of lake water MPBs (Supplementary Figure 3; Supplementary Table 5).

**Figure 4:**
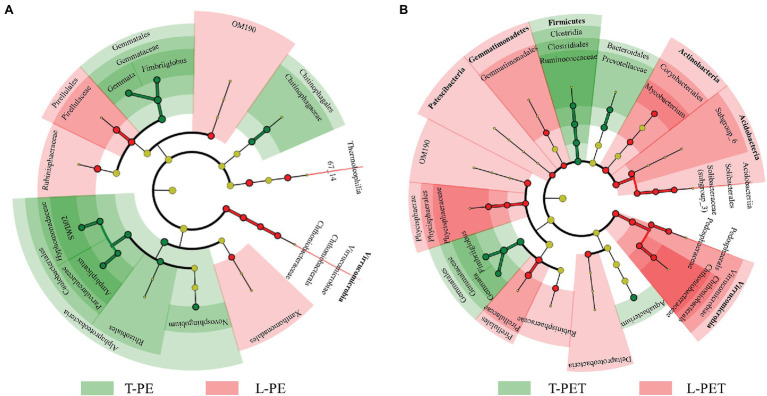
The cladogram of biofilm bacterial clusters with significant dissimilarities (*p* < 0.05) between lake and tap water samples on PE **(A)** and PET **(B)** materials using linear discriminant analysis (LDA) effect size (LEfSe) analysis. The green and red regions (including circles) represent the respective biofilm bacterial clusters originating from tap and lake water, and the yellow circles represent bacterial clusters without significant dissimilarities. Each chart inside out represents biofilm bacterial taxonomies from phylum to genus levels. Bacterial clusters with ≥4 of LDA values are labeled.

The comparison of biomarkers in MPBs with significant dissimilarities between lake and tap water samples was conducted using LEfSe analysis. For the biofilms on PE material, as shown by Figure 4A, tap water MPBs contain more biomarkers (i.e., *Alphaproteobacteria*, *Gemmatales*, and *Chitinophagales*) than lake water MPBs (i.e., Virrucomicrobia, OM190, *Pirellulales*, and *Rubinisphaeraceae*). Furthermore, the genera *SWB02*, *Apolymeric materialshiplicatus*, *Gemmata*, and *Fimbriiglobus* in tap water MPBs and the family *Chthoniobacteraceae* in lake water MPBs were the most important biomarkers, which contributed to the continuous significant dissimilarities for at least three taxonomic levels (i.e., from phylum to genus levels) between L-PE and T-PE (Figure 4A). Moreover, the relative abundance of the bacterial genus *Mycobacterium* showed a significant correlation (*p* < 0.05, Pearson’s *r* = 0.999) between L-PE and T-PE along with the incubation time (Supplementary Table 6). Conversely, concerning the biofilms on PET material, more biomarkers belonged to the MPBs in lake water than in tap water; the families *Solibacteraceae subgroup_3*, *Pedosphaeraceae*, *Chthoniobacteraceae*, and *Phycisphaeraceae* in lake water MPBs, and the family *Ruminococcaceae*, the genera *Gemmata* and *Fimbriiglobus* in tap water MPBs were the most important (Figure 4B). These biomarkers further influenced the functional diversities of MPB communities, of which the genera *Gemmata* and *Pirellula* were reported to be crucial bacteria in nitrogen removal (Xia et al., 2019) and the families *Chthoniobacteraceae* and *Pirellulaceae* are commonly dominant bacteria that play key roles in the interconnection within bacterial communities in lake water (Zhu et al., 2019).

### Co-occurrence Networks Between Bacterial OTUs in Lake and Tap Water MPBs

**Figure 5:**
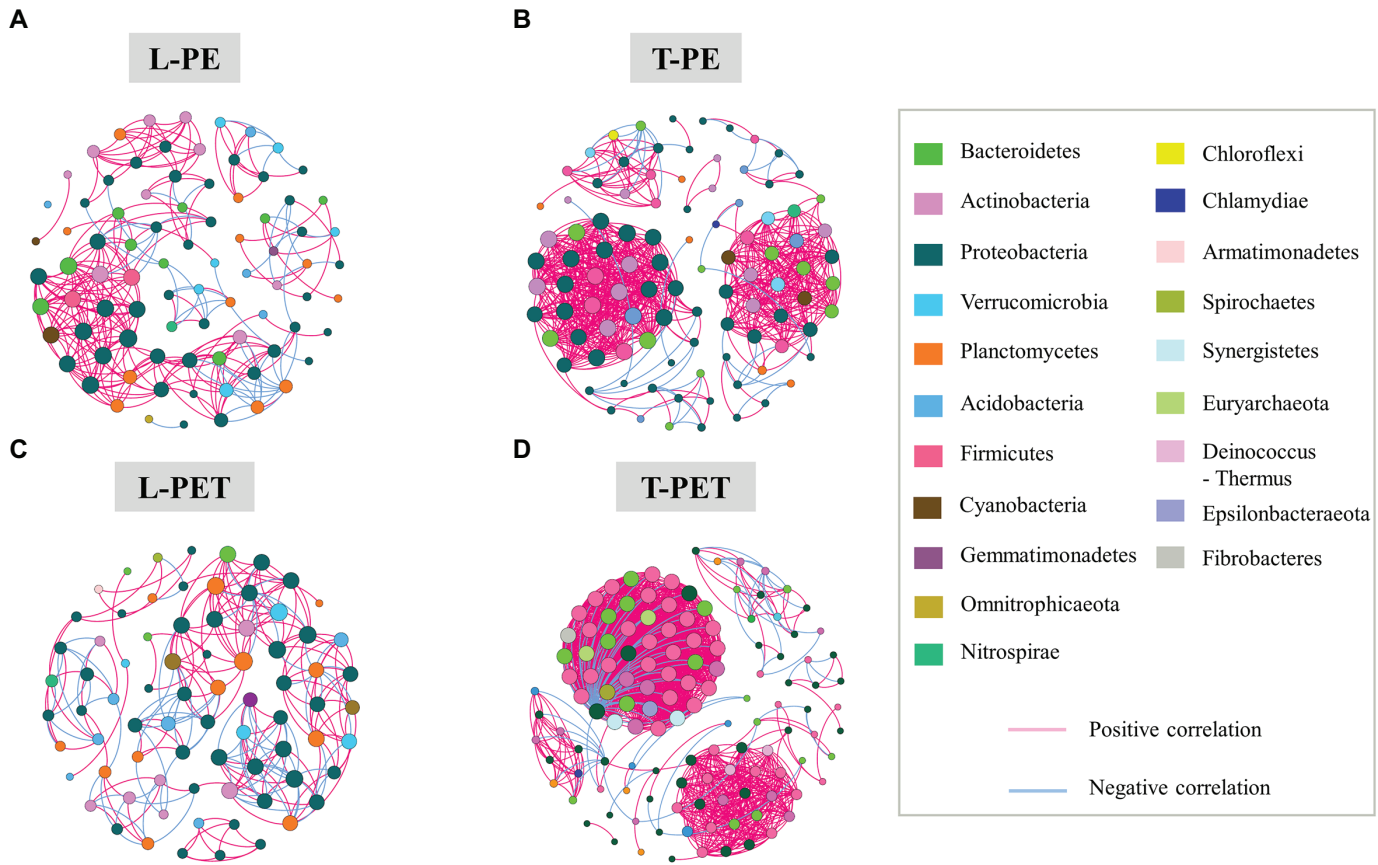
Co-occurrence networks of MPB bacterial communities in L-PE **(A)**, T-PE **(B)**, L-PET **(C)**, and T-PET **(D)** sample groups based on correlation analysis. Each node represents one OTU, of which the size is proportional to the number of connections (i.e., degree) and the color represents bacterial taxonomy at the phylum level. An edge represents significantly strong (*p* < 0.05; Pearson’s correlation coefficient *r* > 0.9) correlation (purple for positive correlation and blue for negative correlation) between bacterial OTUs.

The results presented above identified distinct bacterial diversities (i.e., dynamic succession, structure, and abundance) between MPBs originating from lake and tap water samples, suggesting an underlying discrepancy in the interactions within their complex bacterial communities. Thus, an examination of the co-occurrence networks was carried out to evaluate the bacterial community complexity of different MPBs. Based on the Pearson correlation coefficient, paired bacterial OTUs that were significantly correlated (*p* < 0.05; Pearson’s |*r*| > 0.9) were chosen for further topology analysis using Gephi v 0.9.2 software. In the co-occurrence network, nodes and edges represent taxonomic OTUs at the phylum level and significant correlations between paired OTUs, respectively. Furthermore, colors were used for the classification of different bacterial phyla (for nodes) and positive/negative correlations (for edges), and sizes of nodes are proportional to the degree that characterizes the number of edges. In Figure 5, it can be clearly observed that the number of nodes and edges was dramatically higher for biofilms in the tap water sample (99 and 127 nodes; 685 and 1,575 edges) than for those in the lake water sample (84 and 76 nodes; 287 and 253 edges) irrespective of PE or PET materials, indicating that the bacterial intercorrelations of MPBs in tap water were more complicated. Furthermore, the values of average degree (i.e., the number of edges per node) were higher for T-PE/T-PET (13.838/24.803) than for L-PE/L-PET (6.714/6.658), which may also characterize the complex interconnectivity of the whole tap water MPB networks (Supplementary Table 7). Interestingly, the number of nodes and edges was more likely to be positively correlated with bacterial richness and diversity, which has been proposed in previous studies (Wu et al., 2019; Wang et al., 2020). However, this study’s results showed a lower bacterial OTU number and richness (i.e., Ace and Chao values) for tap water MPBs than for those in lake water.

The modularity parameter was used to determine whether a network had a modular structure, in which case the values should generally exceed 0.4 (Newman, 2006; Barberan et al., 2012). In this study, the modularity parameters of all MPBs (0.656 for L-PE; 0.568 for T-PE; 0.72 for L-PET; and 0.407 for T-PET) indicated that the networks within each biofilm bacterial community had modular structures (Supplementary Table 7). The modularity values of tap water MPBs were lower than those of lake water MPBs, which are generally characterized by low independence among different modular groups. Moreover, the characteristic of the modularity parameter, combined with the average path length and the clustering coefficient, have commonly been used to estimate the degree of interconnection among different members within a network (Zhou et al., 2010; Deng et al., 2012; Wang et al., 2020). Within a biofilm bacterial community, the short average path length and high clustering coefficient meant that the effects of external disturbances could immediately reach to the whole community network, which would be further amplified if different modular groups were highly interdependent (i.e., low modularity; Faust and Raes, 2012; Wu et al., 2019; Wang et al., 2020). Of the MPBs examined in this study, L-PE and L-PET had low clustering coefficients and longer average path lengths compared to those of T-PE and T-PET (Supplementary Table 7), suggesting that the structure and function of lake water MPBs were more stable than tap water MPBs under environmental perturbations. Furthermore, bacterial nodes with a high degree (i.e., connectivity) were generally considered to be critical species sustaining the stability of the entire bacterial community (Berry and Widder, 2014; Shi et al., 2016). Previous studies have frequently identified the phylum Proteobacteria as keystone bacteria in natural MPBs (Wu et al., 2019; Wang et al., 2020), which is consistent with this study’s results for all of the MPBs except T-PET (keystone bacteria: Firmicutes). Moreover, in contrast to lake water MPBs, most of the correlations between paired bacterial OTUs in tap water MPBs were positive, revealing cooperative interrelations among bacterial species in tap water MPBs (Figure 5; Supplementary Table 8).

### Potential Functions of Lake and Tap Water MPBs

**Figure 6:**
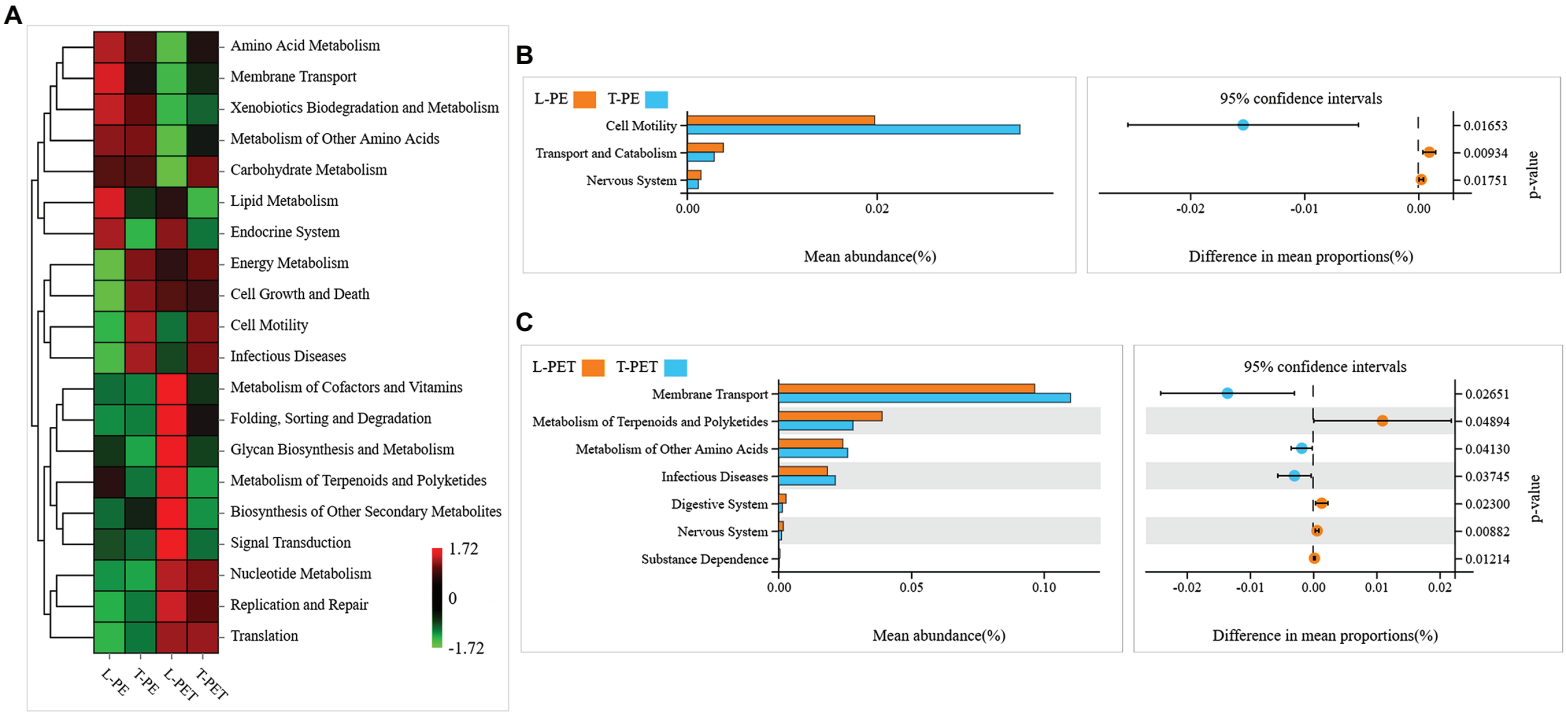
Potential metabolic functions of biofilm bacterial communities on PE and PET materials according to Kyoto Encyclopedia of Genes and Genomes (KEGG) metabolic pathway (level 2). The correlations between each sample group and top 20 metabolic functions in KEGG pathway are shown in heatmap **(A)**. The column and dot plots **(B,C)** show the mean abundance and difference in mean proportions of metabolic functions, which are significantly dissimilar (*p* < 0.05) between two biofilm sample groups using Welch’s *t*-test.

The differences in potential functions of MPBs originating from lake and tap water sample were predicted using bioinformatic tools. In this study, the top 20 metabolic functions based on level 2 of the KEGG metabolic pathway were selected. It was clearly evident that the metabolic functions of T-PE and T-PET were similar, whereas those of L-PE and L-PET were mostly opposite (Figure 6A). Furthermore, the metabolic function of membrane transport was significantly (*p* < 0.05) higher in T-PET than in L-PET (Figure 6C), indicating that the fundamental metabolic activities of PET-biofilm in the tap water sample could be better maintained than those of PET-biofilm in the lake water sample. Moreover, the significantly higher degree of cell motility and membrane transport (Figures 6B,C), which are essential functions for biofilm formation and maturation (Bryant et al., 2016; Jiang et al., 2018), indicated that the MPBs examined in this study were more easily formed in tap water than in lake water.

**Figure 7:**
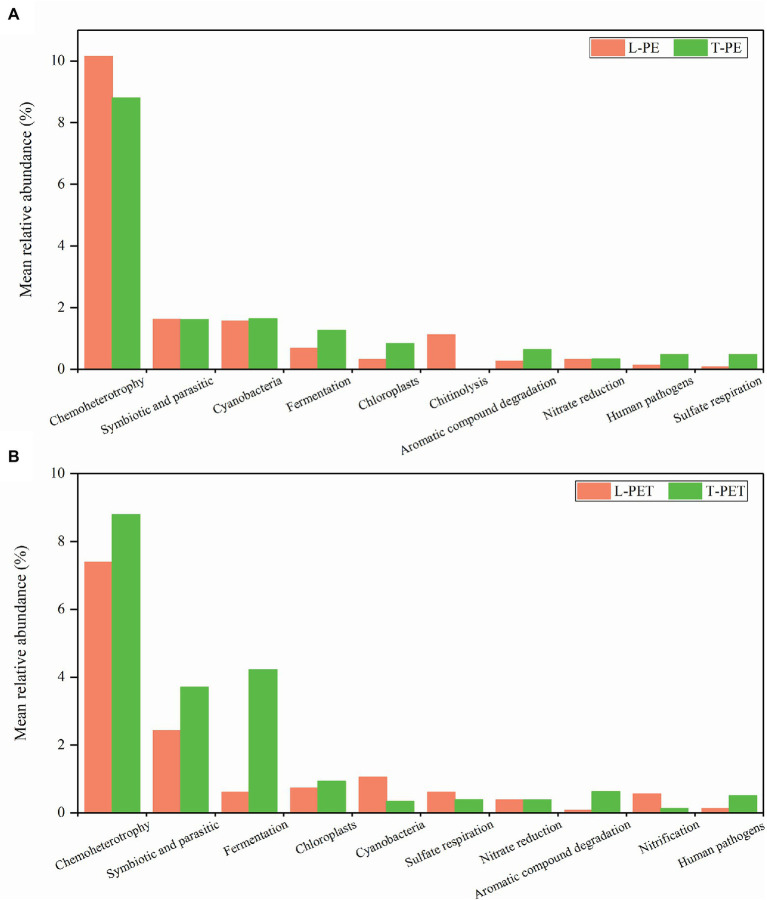
Mean relative abundance of dominant ecological functions in each biofilm sample group. The top 10 ecological functions of biofilm samples on PE **(A)** and PET **(B)** materials are selected, respectively.

In addition, distinct ecological functions were observed in MPBs from lake and tap water samples, which were attributed to dominant and exclusive bacterial groups. Similarly, the highest relative abundance for PE- and PET-biofilms in ecological functions was chemoheterotrophy, followed by symbiotic and parasitic functions (Figure 7). The enrichment of symbionts and parasites on polymeric materials is not surprising and is attributable to the fact that tight cooperations among bacterial species are ever-present within these microecosystems (Wu et al., 2019; Li et al., 2020; Wang et al., 2020). Moreover, different ecological functions at low abundance in PE and PET biofilms (e.g., cyanobacteria, aromatic compound degradation, human pathogens, and sulfur and nitrogen cycle) were mainly attributed to one or several exclusive bacterial taxa as mentioned above, thereby exhibiting distinct ecological functions in different biofilms. Nevertheless, according to the statistical analysis, none of these ecological functions exhibited significant dissimilarities between MPBs from lake and tap water samples, suggesting similar ecological functions of MPBs exposed to different freshwater habitats.

## Conclusion

Microplastics could serve as vectors that selectively enrich distinct bacterial assemblages and associated functions from different freshwater environments. Herein, the dynamic changes of bacterial communities in MPBs reveal that the successions of biofilm communities on different microplastic particles are dissimilar over a long period of incubation in the same freshwater environment, especially in lake water. Furthermore, the MPB communities exhibit distinct interconnective characteristics; that is, the structures of lake water MPBs are more stable under external stresses, and the bacterial species interact more cooperatively in tap water MPBs. Thus, microplastics are likely to be gathering habitats for several symbionts and parasites. More importantly, indispensable functions in biofilm formation and maturation are more significantly encoded in tap water MPBs.

## Data Availability Statement

The original contributions presented in the study are publicly available. This data can be found here: The datasets generated during or analyzed during the current study are available from the corresponding author on reasonable request. The 16S amplicon sequences were available at National Center for Biotechnology Information (NCBI) Sequence Read Archive with the accession numbers from SRR11911199 to 11911214.

## Author Contributions

YS contributed to the study conception and design. Material preparation, data collection and analysis were performed by all authors. The first draft of the manuscript was written by YS and BZ, and all authors commented on previous versions of the manuscript. All authors read and approved the final manuscript.

## Funding

This work was supported by the National Natural Science Foundation of China (no. 32001193 to YS and no. 31971503 to RW) and Shandong Provincial Agricultural Fine Species Project (2019LZGC020 to RW).

## Conflict of Interest

The authors declare that the research was conducted in the absence of any commercial or financial relationships that could be construed as a potential conflict of interest.

## Publisher’s Note

All claims expressed in this article are solely those of the authors and do not necessarily represent those of their affiliated organizations, or those of the publisher, the editors and the reviewers. Any product that may be evaluated in this article, or claim that may be made by its manufacturer, is not guaranteed or endorsed by the publisher.
