# Supplementary material for: Comparative Analysis of Selective Bacterial Colonization by Polyethylene and Polyethylene Terephthalate Microplastics
Source: Front Microbiol. 2022 Feb 2;13:836052. doi: 10.3389/fmicb.2022.836052 (PMC8847747; doi:10.3389/fmicb.2022.836052)
Supplement: Supplementary file 1 [file Data_Sheet_1.docx]

Supplementary Material

**Comparative analysis of selective bacterial colonization by polyethylene and polyethylene terephthalate microplastics**

**Yuhao Song*, Baoxin Zhang, Lianwei Zou, Feng Xu, Yaqi Wang, Shaoqi Xin, Yang Wang, Hongyuan Zhang, Ning Ding and Renjun Wang***

College of Life Sciences, Qufu Normal University, Qufu, China.

**The experimental method of carbon-free glassware preparation:**

All glassware used in this study was pre-treated to ensure they were carbon free. In brief, bottles and vials were washed three times with detergent and rinsed with ultrapure water, then soaked overnight in 0.2 M HCl solution to remove any inorganic substances. Afterwards, glassware was washed with ultrapure water, air-dried, and heated in a muffle furnace at 550 °C for 5 h before use. Meanwhile, the screw caps were rinsed and soaked in 10% sodium persulfate solution at 60 °C for 1 h, then washed three times with ultrapure water and air-dried before use.

**Supplementary Table 1.** Water quality parameters of two freshwater samples (n = 3, standard deviation < 5%)

| Item (unit) | Lake water | Tap water |
| --- | --- | --- |
| pH | 8.17 | 7.62 |
| DO (mg/L) | 8.50 | 8.50 |
| Turbidity (NTU) | 12.6 | ＜0.500 |
| Conductivity  (μs/cm) | 325 | 304 |
| TOC (mg/L) | 6.00 | 2.20 |
| TN (mg/L) | 1.08 | 1.27 |
| NH_3_ – N (mg/L) | 0.208 | ＜0.025 |
| KTN (mg/L) | 1.10 | ＜0.01 |
| TP (mg/L) | 0.07 | ＜0.01 |
| Phosphate (mg/L) | 0.03 | ＜0.01 |
| Biomass (cells/mL) | 8.86 × 10^6^ | 1.12 × 10^5^ |

**Supplementary Table 2.** Good’s coverage values of biofilm bacterial samples

| Bacterial sample | Good’ coverage value |
| --- | --- |
| L-PE-0  L-PE-10  L-PE-20  L-PE-30  L-PET-0  L-PET-10  L-PET-20  L-PET-30  T-PE-0  T-PE-10  T-PE-20  T-PE-30  T-PET-0  T-PET-10  T-PET-20  T-PET-30 | 0.993001996  0.996574915  0.996243449  0.995924379  0.99312483  0.995718713  0.994701147  0.996123214  0.994221794  0.996026209  0.996263038  0.996055599  0.993968557  0.995934918  0.996286569  0.996512005 |

**Supplementary Table 3.** Intrinsic and overlapped OTU number of biofilm bacterial samples on MPs

| Time (d) | L-PE vs. T-PE | | | L-PET vs. T-PET | | |
| --- | --- | --- | --- | --- | --- | --- |
|  | Intrinsic  (L-PE) | Overlapped | Intrinsic  (T-PE) | Intrinsic  (L-PET) | Overlapped | Intrinsic  (T-PET) |
| 10 | 997 | 506 | 443 | 1496 | 541 | 438 |
| 20 | 951 | 515 | 608 | 1787 | 566 | 486 |
| 30 | 782 | 607 | 782 | 1555 | 550 | 677 |

**Supplementary Table 4.** Alpha diversity indices of biofilm bacterial samples

| Biofilm samples | Alpha diversity indices | | | |
| --- | --- | --- | --- | --- |
|  | Shannon | Chao | Ace | Simpson |
| L-PE-0 | 5.908493 | 2067.235 | 2241.988 | 0.92274 |
| L-PE-10 | 7.720462 | 1885.807 | 1898.68 | 0.984769 |
| L-PE-20 | 7.523336 | 1871.918 | 1893.414 | 0.982589 |
| L-PE-30 | 6.809093 | 1954.319 | 1947.067 | 0.943443 |
| L-PET-0 | 5.953184 | 1943.189 | 2122.608 | 0.938015 |
| L-PET-10 | 8.256985 | 2417.193 | 2350.805 | 0.990158 |
| L-PET-20 | 8.688225 | 2692.373 | 2710.213 | 0.993099 |
| L-PET-30 | 8.50867 | 2395.254 | 2399.112 | 0.990894 |
| T-PE-0 | 7.139593 | 2078.799 | 2182.075 | 0.97723 |
| T-PE-10 | 6.551246 | 1523.836 | 1598.367 | 0.975716 |
| T-PE-20 | 7.120426 | 1702.863 | 1752.491 | 0.98537 |
| T-PE-30 | 7.84972 | 1962.375 | 2027.32 | 0.990567 |
| T-PET-0 | 7.05028 | 2322.453 | 2427.947 | 0.971126 |
| T-PET-10 | 6.409154 | 1588.045 | 1614.706 | 0.969899 |
| T-PET-20 | 6.599186 | 1544.086 | 1710.575 | 0.968604 |
| T-PET-30 | 8.260757 | 1612.595 | 1709.193 | 0.993464 |

**Supplementary Table 5.** Relative abundance of dominant biofilm bacteria at the phylum, class, and genus levels in biofilm sample groups

| Biofilm bacteria | Relative abundance (%) | | | |
| --- | --- | --- | --- | --- |
|  | L-PE | T-PE | L-PET | T-PET |
| **Phylum** |  |  |  |  |
| Proteobacteria | 33.10 | 46.27 | 26.10 | 28.90 |
| Planctomycetes | 26.4137 | 31.71433 | 30.05283 | 39.23353 |
| Actinobacteria | 15.84 | —— | —— | —— |
| Bacteroidetes | —— | 7.00 | —— | —— |
| Acidobacteria | —— | —— | 7.62 | —— |
| Firmicutes | —— | —— | —— | 12.04 |
| **Class** |  |  |  |  |
| *Planctomycetacia* | 18.84 | 31.20 | 18.18 | 38.97 |
| *Gammaproteobacteria* | 18.80 | 20.46 | 10.84 | 16.75 |
| *Alphaproteobacteria* | —— | 23.85 | 9.45 | 11.20 |
| *Thermoleophilia* | 11.44 | —— | —— | —— |
| **Genus** |  |  |  |  |
| *Aquabacterium* | 2.85 | 11.79 | —— | 10.03 |
| *Fimbriiglobus* | —— | 4.73 | —— | 6.94 |
| *Gemmata* | —— | 5.46 | —— | 2.64 |
| *Pirellula* | 2.58 | —— | 3.38 | —— |
| *Mycobacterium* | —— | —— | 2.67 | —— |
| *Chthoniobacter* | 2.14 | —— | 1.70 | —— |

* The relative abundance of top 3 bacteria in each sample group are listed.

**Supplementary Table 6.** Relative abundance of biofilm bacterial genus *Mycobacterium* on PE materials within the 30 days of incubation in lake and tap water (*P* < 0.05, Pearson’s r = 0.999)

| Sample | Relative abundance | | |
| --- | --- | --- | --- |
|  | 10 d | 20 d | 30 d |
| L-PE | 1.74 % | 1.75 % | 2.23 % |
| T-PE | 0.27 % | 0.31 % | 1.33 % |

**Supplementary Table 7.** Main topology parameters of the co-occurrence networks calculated by Gephi

| Topology parameters | L-PE | T-PE | L-PET | T-PET |
| --- | --- | --- | --- | --- |
| Number of nodes | 84 | 99 | 76 | 127 |
| Number of edges | 287 | 685 | 253 | 1575 |
| Modularity | 0.656 | 0.568 | 0.72 | 0.407 |
| Network diameter | 9 | 7 | 19 | 4 |
| Average path length | 2.956 | 2.105 | 6.054 | 1.191 |
| Average degree | 6.714 | 13.838 | 6.658 | 24.803 |
| Average clustering coefficient | 0.78 | 0.889 | 0.758 | 0.927 |

**Supplementary Table 8.** Number of paired bacterial OTUs with significantly positive and negative Pearson’ correlations at the phylum, class, and genus levels in each biofilm sample group

| Biofilm sample group |  | Paired bacterial species number |  |
| --- | --- | --- | --- |
| (Taxonomic level) | Total | Positive | Negative |
| **L-PE** |  |  |  |
| (Phylum) | 5 | 4 | 1 |
| (Class) | 63 | 45 | 18 |
| (Genus) | 287 | 235 | 52 |
| **T-PE** |  |  |  |
| (Phylum) | 6 | 5 | 1 |
| (Class) | 20 | 17 | 3 |
| (Genus) | 685 | 645 | 40 |
| **L-PET** |  |  |  |
| (Phylum) | 10 | 5 | 5 |
| (Class) | 67 | 40 | 27 |
| (Genus) | 253 | 162 | 91 |
| **T-PET** |  |  |  |
| (Phylum) | 18 | 11 | 7 |
| (Class) | 55 | 48 | 7 |
| (Genus) | 1575 | 1484 | 91 |


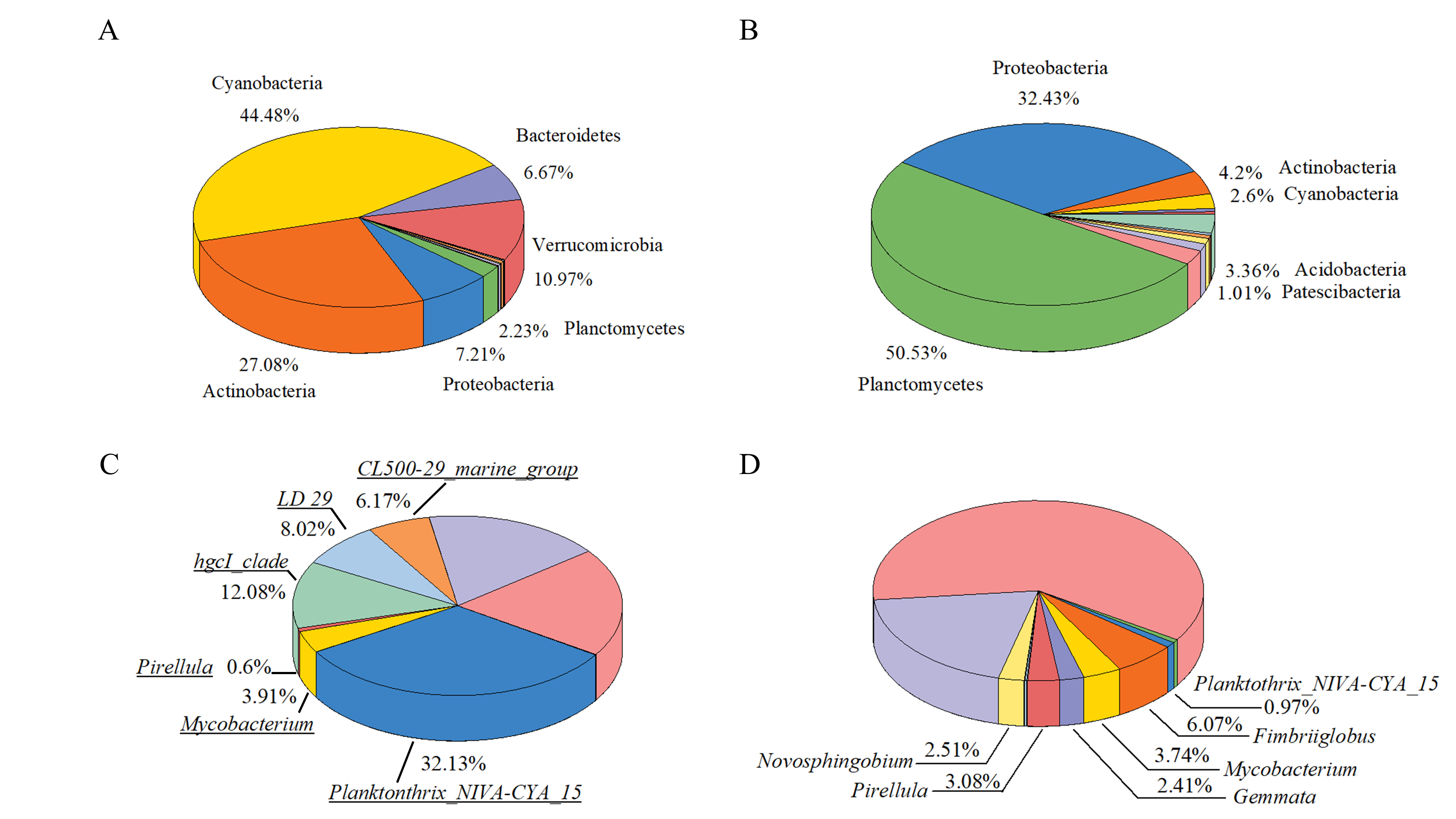


**Supplementary Figure 1.** Relative abundance of dominant bacterial taxonomies in the sampling lake (A and C) and tap (B and D) water samples at the phylum (A and B) and genus (C and D) levels.


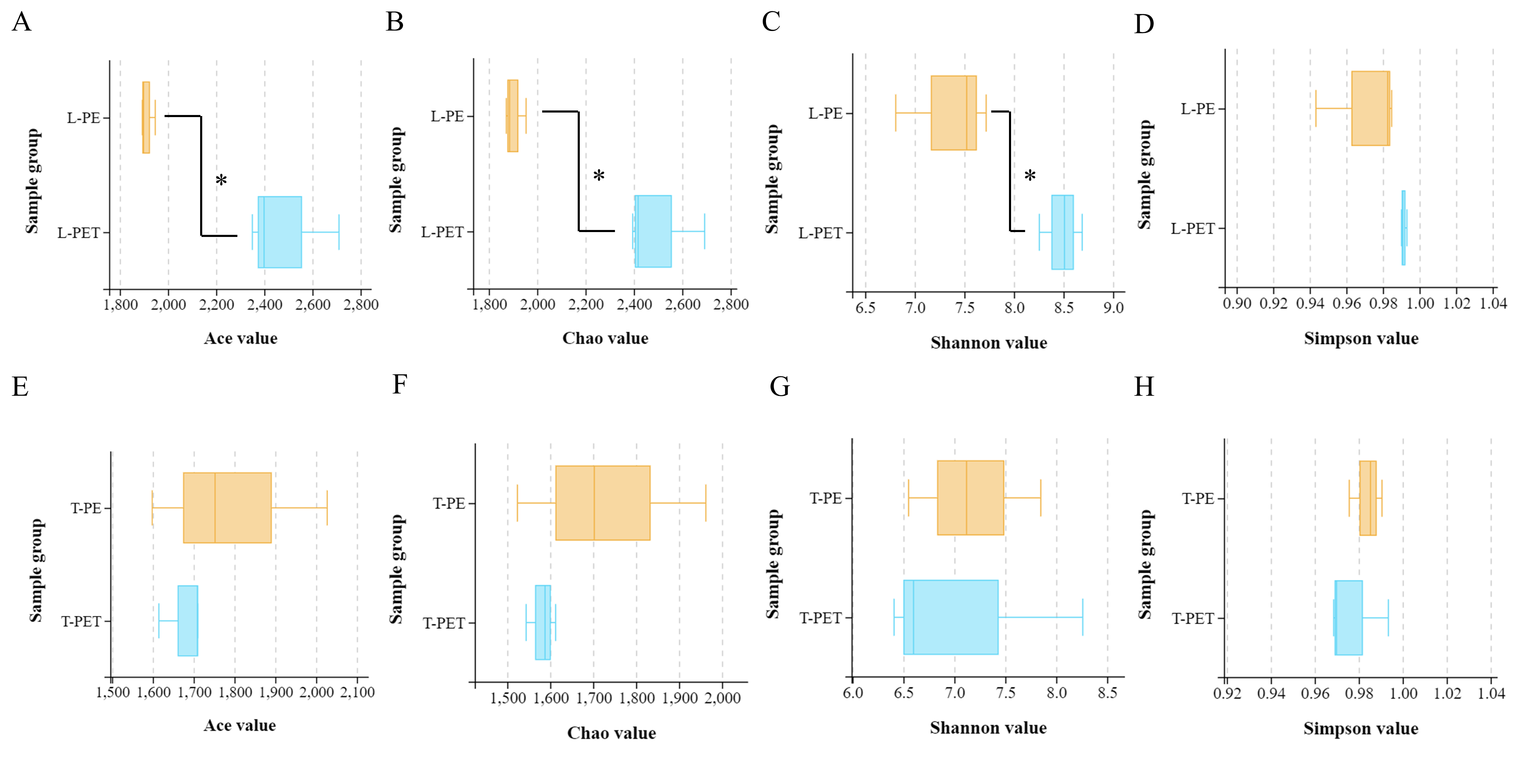


**Supplementary Figure 2.** Box plots showing the Ace, Chao, Shannon, and Simplon indexes related to alpha diversity of different biofilm sample groups. The asterisk (*) represent significant (*P* < 0.05) dissimilarities between two biofilm sample groups.


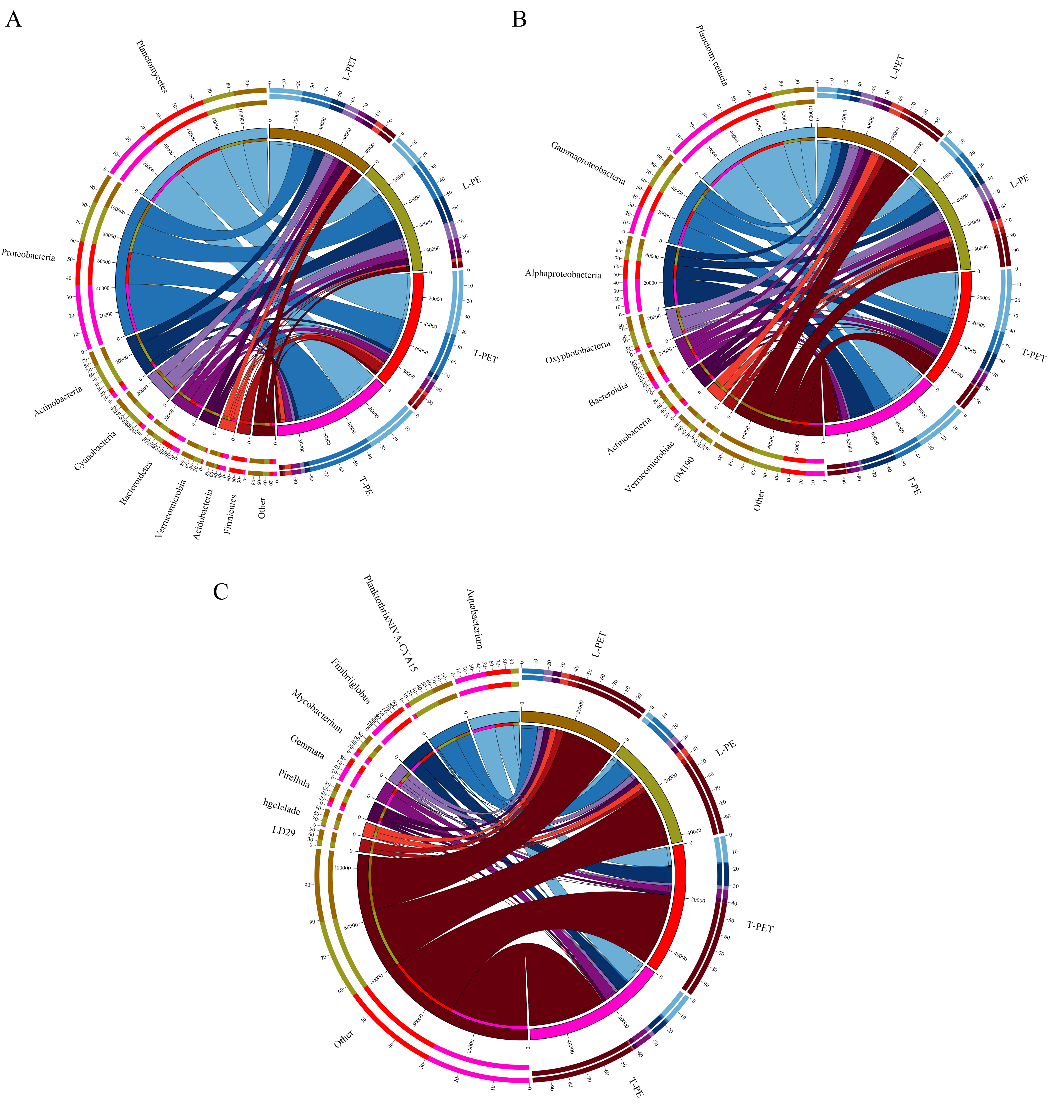


**Supplementary Figure 3.** Relative abundance of dominant MPB bacterial taxonomies in each sample group at the phylum (A), class (B), and genus (C) levels.
